# Tracking daily fatigue fluctuations in multiple sclerosis: ecological momentary assessment provides unique insights

**DOI:** 10.1007/s10865-017-9840-4

**Published:** 2017-03-09

**Authors:** Daniel J. H. Powell, Christina Liossi, Wolff Schlotz, Rona Moss-Morris

**Affiliations:** 1 Aberdeen Health Psychology Group, Institute of Applied Health Sciences, University of Aberdeen, 2nd Floor Health Sciences Building, Foresterhill Campus, Aberdeen, AB25 2ZD UK; 2 Psychology, Faculty of Social and Human Sciences, University of Southampton, Southampton, SO17 1BJ UK; 3 Max Planck Institute of Empirical Aesthetics, 60322 Frankfurt am Main, Germany; 4 Health Psychology Section, Psychology Department, Institute of Psychiatry, Psychology and Neuroscience, Kings College London, London, SE1 9RT UK

**Keywords:** Multiple sclerosis, Fatigue, Ecological momentary assessment, Ambulatory assessment, Psychological stress, Affect

## Abstract

Studies investigating the prevalence, cause, and consequence of multiple sclerosis (MS) fatigue typically use single measures that implicitly assume symptom-stability over time, neglecting information about *if*, *when*, and *why* severity fluctuates. We aimed to examine the extent of moment-to-moment and day-to-day variability in fatigue in relapsing-remitting MS and healthy individuals, and identify daily life determinants of fluctuations. Over 4 weekdays, 76 participants (38 relapsing-remitting MS; 38 controls) recruited from multiple sites provided real-time self-reports six times daily (*n* = 1661 observations analyzed) measuring fatigue severity, stressors, mood, and physical exertion, and daily self-reports of sleep quality. Fatigue fluctuations were evident in both groups. Fatigue was highest in relapsing-remitting MS, typically peaking in late-afternoon. In controls, fatigue started lower and increased steadily until bedtime. Real-time stressors and negative mood were associated with increased fatigue, and positive mood with decreased fatigue in both groups. Increased fatigue was related to physical exertion in relapsing-remitting MS, and poorer sleep quality in controls. In relapsing-remitting MS, fatigue fluctuates substantially over time. Many daily life determinants of fluctuations are similar in relapsing-remitting MS and healthy individuals (stressors, mood) but physical exertion seems more relevant in relapsing-remitting MS and sleep quality most relevant in healthy individuals.

**Electronic supplementary material:**

The online version of this article (doi:10.1007/s10865-017-9840-4) contains supplementary material, which is available to authorized users.

## Introduction

Approximately 65–80% of people with multiple sclerosis (MS) experience severe fatigue (Hadjimichael et al., 2008; Lerdal et al., 2003; Minden et al., 2006). Fatigue is usually assessed in research and clinical practice by asking patients to provide recalled summaries of severity or impact over a period of time (Tyson & Brown, 2014). However, this implicitly assumes symptom-constancy over the time period, overlooking potentially important information about day-to-day, moment-to-moment, and context-dependent fluctuations. We present the first prospective quantitative study to determine the extent of within-person fatigue fluctuations in MS in daily life, examining temporal and contextual determinants of fatigue severity in people with relapsing-remitting MS and healthy individuals in daily life.

MS fatigue is commonly defined as “a subjective lack of physical and/or mental energy that is perceived by the individual or caregiver to interfere with usual and desired activities” (Multiple Sclerosis Council for Clinical Practice Guidelines, 1998, p. 2). Fatigue is considered a subjective sensation, with objective changes in mental or physical performance conceptualized as fatigability (Kluger et al., 2013). The pathology of MS fatigue is poorly understood, and fatigue is commonly thought to emanate from both primary (centrally-mediated disease factors) and secondary (all other factors) sources (Kos et al., 2008). Neurological symptoms, depressive symptoms, and sleep disturbance have been found to independently contribute to overall variance in fatigue in MS (Strober & Arnett, 2005) and others have noted multiple other sources of fatigue in MS, including psychosocial stress, unhealthy lifestyles, and physical exertion (Mills & Young, 2008).

Initial insights into the everyday dynamics of MS fatigue have implied a fluctuating symptom, with qualitative and, small clinic-based, quantitative studies suggesting fatigue is typically worst in the latter part of the day (Claros-Salinas et al., 2010; Feys et al., 2012; Freal et al., 1984; Mills & Young, 2008; Morris et al., 2002) and is exacerbated by psychosocial stress (Mollaoğlu & Üstün, 2009; Stuifbergen & Rogers, 1997). It remains unclear whether fatigue in MS has a unique pattern of relationships with stress and mood-disturbance, or whether it mirrors associations also found in healthy individuals (Gledhill, 2005). Although physical (in)activity and sleep are considered relevant to MS fatigue (Strober, 2015; Stroud & Minahan, 2009) the immediacy of their effects is poorly understood.

The present study investigated day-to-day and moment-to-moment fluctuations in fatigue severity in people with relapsing-remitting MS and healthy individuals. Based on previous studies, we expected fatigue to vary significantly within-individuals in relapsing-remitting MS. Controlling for baseline depressive symptoms and chronic stress, we expected fatigue severity to increase across the day in relapsing-remitting MS, at a faster rate than in controls. We also expected fatigue to vary within-individuals, in both groups, with poorer sleep quality, physical exertion, psychosocial stress, and negative mood (independent of positive mood), whilst varying inversely with positive mood (independent of negative mood).

## Method

This article presents a first analysis of real-time self-report data collected within an investigation of associations between cortisol and fatigue in relapsing-remitting MS, published elsewhere (Powell et al., 2015).

### Participants

Between February 2012 and February 2013, 42 people with clinically-definite relapsing-remitting MS (Polman et al., 2011) as determined by a neurologist, and 40 healthy individuals well-matched for age and gender were recruited. Eligibility criteria are outlined in Table 1. The relapsing-remitting MS group was recruited from multiple sites: consecutive eligible patients at neurologist and specialist nurse clinics at University Hospital Southampton NHS Foundation Trust and Guy’s and St Thomas’ NHS Foundation Trust, and nearby MS Society networks. Once an individual was recruited to the MS group, an individual of the same gender and similar age (±3 years) was recruited to the healthy control group from the local community (Hampshire and Greater London). Of those patients referred to participate in the study, 76 of 205 (37%) were eligible, of which 42 (55%) took part. The control group (40 of 55 invited; 72%) was recruited from local postings in Hampshire and Greater London. Data from four participants were lost to technical faults or a discovered endocrine abnormality, and two participants withdrew prior due to unrelated illness or personal reasons, leaving 38 individuals in each group.

**Table 1:** Participant recruitment eligibility criteria

Relapsing-remitting MS group	Healthy control group
Inclusion criteria: A clinically-definite diagnosis of relapsing-remitting MS (Polman et al., 2011) Aged 18–65 years	Inclusion criteria Healthy individual Aged 18–65 years
Exclusion criteria: A recent (within 3 months) clinical relapse or corticosteroid treatment An inability to ambulate 300 metres without rest An additional physical or psychiatric diagnosis A high level of depressive symptoms [scoring ≥ 8 on the depression subscale of the Hospital Anxiety and Depression Scale (Zigmond & Snaith, 1983)] Current prescription of antidepressant medication Currently pregnant Shift-worker Caregiver	Exclusion criteria: A current chronic or acute disease or illness A current prescription for any medication Currently pregnant Shift-worker Caregiver

Ethical approval was granted by the UK NHS National Research Ethics Service Committee (11/SC/0333) and the University of Southampton Psychology Ethics Committee. All data included in this manuscript were obtained in compliance with University of Southampton regulations and the Helsinki Declaration. All participants gave written informed consent and, upon completion of the study, received £40 reimbursement for their time and expenses.

### Baseline and training

One-to-one introductory sessions with the researcher took place at the University of Southampton or King’s College London. Here, participants provided demographic information, completed baseline questionnaires, and received training in the electronic handheld device used to prompt the ecological momentary assessment schedule (Shiffman et al., 2008).

### Ecological momentary assessment schedule

Ecological momentary assessment is defined as the relatively intensive and repeated assessment of variables in real-time, in the real-world, as individuals go about their usual daily activities (Shiffman et al., 2008). Ecological momentary assessment was used to collect repeated real-time measurements of fatigue severity and psychosocial determinants, over time, in daily life. Ecological momentary assessment was delivered via handheld device (Hewlett Packard iPAQ 111 Classic Handheld) using software programmed with Microsoft Visual Studio. Over 4 consecutive weekdays, real-time self-reports were prompted by auditory alarm six times per day between 10 am and 8 pm by an algorithm randomly assigning a single prompt within each of six consecutive 100-min periods, with inter-prompt periods of at least 30 min. Participants could postpone responses for 5, 10, or 15 min, and select a silent mode if required. The quasi-random design limits the biases associated with fixed time designs ensuring a representative sample of daily life. A final auditory prompt at 9 pm requested a recall measure.

### Measures

#### Baseline measures

##### Fatigue severity

Participants completed the 11-item Chalder Fatigue Questionnaire (Chalder et al., 1993) which is considered a valid and reliable measure of fatigue severity in MS (Chilcot et al., 2015). Chalder Fatigue Questionnaire scores range from 0 to 33, with higher scores indicate greater fatigue severity over the last month (present study Cronbach *α* = .65).

##### Covariates

Participants completed the 7-item depression subscale from the Hospital Anxiety and Depression Scale (Zigmond & Snaith, 1983) which has been shown to be a valid measure for depression in MS (Honarmand & Feinstein, 2009). Higher subscale scores (possible range: 0–21) indicate high levels of depressive symptoms over the prior week (*α* = .65). The 12-item Chronic Stress Screening Scale (Schulz et al., 2004) was completed, with higher scores indicating greater chronic stress over the previous 3 months (*α* = .91). Chronic Stress Screening Scale scores range from 0 to 48, and this is the first time this measure has been used in MS. Neurological disability in MS was measured by the self-administered Expanded Disability Status Scale (Bowen et al., 2001) incorporating a series of bespoke items covering a spectrum of functioning. Expanded Disability Status Scale scores range from 0 to 10, with higher scores indicating greater disability. The self-administered Expanded Disability Status Scale correlates highly with the physician-delivered Expanded Disability Status Scale (Kurtzke, 1983).

#### Ecological momentary assessment measures

##### Momentary fatigue severity

All ecological momentary assessment measures are shown in full in Supplementary Materials 1. Real-time *Momentary Fatigue Severity* was measured by a single item: ‘How much fatigue (tiredness, weariness, problems thinking clearly) do you feel right now?’ with responses from 0 ‘None at all’ to 10 ‘Extreme Fatigue’. This item was based on the ‘Right Now’ item from the Brief Fatigue Inventory (Mendoza et al., 1999) with ‘problems thinking clearly’ added to reflect mental fatigue (Multiple Sclerosis Council for Clinical Practice Guidelines, 1998). Convergent validity was demonstrated by strong, negative, within-person associations with ‘Energetic’ (*γ* = −0.53, *p* < .001) and ‘Alert’ (*γ* = −0.47, *p* < .01) items, and discriminant validity by weak associations with ‘Anxious’ (*γ* = 0.18, *p* = .33) and ‘Distressed’ (*γ* = 0.08, *p* = .74) items.

##### Momentary stressor exposure

Eight items assessing real-time daily life stress were based on domains of the Trier Inventory for Chronic Stress (Schulz et al., 2004). All items (e.g., ‘I did a lot of work’) were prefixed by ‘Since the last event…’ (i.e., last alarm) and responses were from 0 ‘Not at all’ to 10 ‘Very much so’. An exploratory factor analysis found three factors (Supplementary Materials 2). Due to the limited within-subject reliabilities (Geldhof et al., 2014) of these factor scores identified by the factor analysis, these were discarded in favor of testing the unique effects of each stressor item in exploratory models.

##### Momentary mood

Fifteen mood adjectives (e.g., ‘Irritable’) used in a previous study by our research group (Powell & Schlotz, 2012) were prefixed by ‘At the moment, I feel…’ with responses from 0 ‘Not at all’ to 10 ‘Very much so’. An exploratory factor analysis (Supplementary Materials 2) yielded two independent factors: *Negative Mood* (10 items) and *Positive Mood* (5 items). Scale scores were computed as the mean of items and demonstrated satisfactory within-subject reliabilities (*NA*: *ω*_within_ = .86; *PA*: *ω*_within_ = .68).

##### Daily life behaviors

Participants provided real-time self-reports, prefixed by ‘In the last 30 min…’, for physical exertion, napping, smoking, having a meal, and drinking coffee (‘yes’/‘no’ responses). Sleep quality was rated upon awakening by ‘How would you rate the quality of your sleep last night?’ from 0 ‘Very bad’ to 10 ‘Very good’.

##### Daily fatigue severity

Recalled *Daily Fatigue Severity* was measured at 9 pm by a single item: ‘How much fatigue (tiredness, weariness, problems thinking clearly) have you felt today?’ with responses from 0 ‘None at all’ to 10 ‘Extreme Fatigue’.

### Statistical analysis

Group comparisons for baseline measures and person-mean real-time and daily assessments used t-tests and Mann–Whitney U tests. Person-mean refers to the mean average for a single individual. A bivariate Spearman’s rank correlation matrix examined the relatedness of the different temporal measures of fatigue severity (Chalder Fatigue Questionnaire, person-mean *Daily Fatigue Severity*, person-mean *Momentary Fatigue Severity*) and their respective associations with Depression subscale, Chronic Stress Screening Scale, and Expanded Disability Status Scale.

To appropriately test our main hypotheses, 3-level multilevel models were used that nested *Momentary Fatigue Severity* assessments within days, within individuals. Multilevel modelling was deemed most appropriate as it accounts for nested data and permits unequally spaced assessments, whilst robust to missing data (Black et al., 2012; Singer & Willett, 2003). The models used maximum likelihood estimation to account for missing data that showed no discernable pattern, suggesting these data were missing at random. Null model residuals indicated the proportion of the overall variability in fatigue that was attributable to each of the three levels: moment-to-moment fluctuations, day-to-day fluctuations, and individual differences. Diurnal fatigue patterns (the typical pattern over time for each group) were assessed by adding linear and quadratic fixed and random time effects, with fixed group and group-by-time interaction effects. Potential covariates (napping, smoking, caffeine, age, gender) were tested, with statistically significant covariates retained in the final model. Fixed effects of the Depression subscale and Chronic Stress Screening Scale scores were entered into final models (Model A; Supplementary Materials 3) with Expanded Disability Status Scale score also entered where the relapsing-remitting MS group was comparator.

In order to test the effects of mood and stress, several models were run, based on Model A, with real-time predictors (behaviors, stressors, mood) added as fixed effects with interactions with group to detect group differences in their effects (Models B–D; see Supplementary Materials 3). Model B included physical exertion and sleep quality as predictors; Model C, the eight stressor items; and Model D, the two mood factors. Random effects of statistically significant predictors were then entered into each model to test whether these effects varied substantially across people. In all models, baseline predictors were centered about the grand-mean (i.e., extent an individual scored above/below the average level across all participants). Real-time covariates and predictors were person-mean centered for within-person analysis (i.e., extent a real-time rating was above/below an individual’s usual level). Time was centered at 10am. Analyses used SPSS Version 23. The criterion for statistical significance was *α* = .05.

## Results

Analysis was based on 1661 completed assessments (90.9% of scheduled in the relapsing-remitting MS group; 91.2% in control group) across 304 days, within 76 participants (38 relapsing-remitting MS; 38 control). Table 2 shows group comparisons for baseline and ecological momentary assessment measures. The relapsing-remitting MS group had higher average *Momentary Fatigue Severity*, *d* = 1.30, 95% CI [0.80, 1.79] and *Daily Fatigue Severity*, *d* = 1.44, 95% CI [0.93, 1.93] than the control group. The person-means of two types of stressor (*Excessive Demands*; *Social Isolation*) and *Negative Mood* were significantly higher in the relapsing-remitting MS group. *Positive Mood* was only marginally lower in the relapsing-remitting MS group.

**Table 2:** Demographic and clinical characteristics of recruited sample

Relapsing-remitting MS		Control	*p*
*n*	38	38	
Age	41.89 (7.53)	40.34 (8.16)	
Gender	31F	31F	
*Employment*			
Paid employment	30	33	
Unpaid employment	3	1	
Unemployed	5	4	
Expanded Disability Status Scale	4.29 (1.37)		
Years since diagnosis	6.03 (5.18)		
*Disease modifying therapy (DMT)*			
Interferon	12		
Glatiramer acetate	6		
Natalizumab	5		
No DMT	15		
HADS-depression	4.00 (2.29)	2.08 (2.27)	<.001
Chronic Stress Screening Scale	19.82 (9.36)	14.11 (7.93)	.006
Chalder Fatigue Questionnaire	17.58 (7.09)	11.55 (2.87)	<.001
Ecological Momentary Assessments (average of person-means)			
*Fatigue severity*			
Momentary fatigue severity	5.07 (2.30)	2.42 (1.72)	<.001
Daily fatigue severity	4.74 (2.27)	1.80 (1.77)	<.001
*Stressor exposure*			
Work overload	4.55 (1.45)	5.20 (1.80)	.098
Social overload	4.32 (2.26)	4.02 (2.04)	.68
Excessive demands at work	2.00 (1.31)	1.31 (1.22)	.023
Lack of social recognition	1.98 (1.87)	1.15 (1.33)	.050
Work discontent	2.70 (1.96)	2.78 (2.04)	.86
Social tensions	0.95 (1.02)	0.70 (0.88)	.33
Pressure to perform	3.93 (1.87)	3.45 (2.49)	.39
Social isolation	6.62 (2.25)	5.42 (2.30)	.018
*Mood*			
Negative mood	2.12 (1.15)	1.48 (1.35)	.008
Positive mood	4.91 (1.60)	5.65 (1.63)	.079
*Behavioural*			
Sleep quality	6.07 (1.57)	6.22 (1.97)	.72
Physical exertion (*n* reported bouts daily)	0.45 (0.69)	0.32 (0.48)	.30

Mean (*SD*) shown for all continuous variables. HADS indicates Hospital Anxiety and Depression Scale

Table 3 shows high correlations between person-mean *Momentary Fatigue Severity* and *Daily Fatigue Severity* in both the relapsing-remitting MS group and control group. Person-mean *Momentary Fatigue Severity* and *Daily Fatigue Severity* had the strongest correlations with the Chalder Fatigue Questionnaire in the relapsing-remitting MS group whilst not statistically significant in the control group.

**Table 3:** Nonparametric bivariate correlation matrix of fatigue severity measures, momentary mood, depressive symptoms, chronic stress, and neurological symptoms in people with relapsing-remitting MS and healthy controls

			Fatigue severity measures						Momentary mood (diary)				Baseline covariates			
			MomFS^a^		DailyFS^a^		CFQ		NM^a^		PM^a^		HADS-D		CSSS	
	Mean	SD	*r*_s_	*p*	*r*_s_	*p*	*r*_s_	*p*	*r*_s_	*p*	*r*_s_	*p*	*r*_s_	*p*	*r*_s_	*p*
*Relapsing*-*remitting MS group*																
MomFS^a^	5.07	2.30														
DailyFS^a^	4.74	2.27	.782	<.001												
CFQ	17.58	7.09	.540	<.001	.559	<.001										
NM^a^	2.12	1.15	.290	.078	.320	.050	.148	.37								
PM^a^	4.91	1.60	−.376	.020	−.375	.020	−.233	.16	−.228	.17						
HADS-D	4.00	2.29	.164	.33	.283	.085	.086	.61	.093	.58	−.267	.11				
CSSS	19.82	9.36	.394	.014	.372	.022	.077	.65	.242	.14	−.246	.14	.141	.40		
EDSS^b^	4.29	1.37	.356	.028	.420	.009	.327	.045	.194	.24	−.470	.003	.421	.009	.318	.051
*Control group*																
MomFS^a^	2.42	1.72														
DailyFS^a^	1.80	1.77	.764	<.001												
CFQ	11.55	2.87	.319	.051	.248	.14										
NM^a^	1.48	1.35	.643	<.001	.769	<.001	.258	.12								
PM^a^	5.65	1.63	−.476	.002	−.447	.006	−.169	.31	−.498	.001						
HADS-D	2.08	2.27	.140	.40	.276	.098	.205	.22	.320	.050	−.068	.69				
CSSS	14.11	7.93	.346	.033	.335	.043	.075	.65	.340	.037	−.012	.94	.476	.003		

*MomFS* Momentary Fatigue Severity; *DailyFS* Daily Fatigue Severity; *CFQ* Chalder Fatigue Questionnaire (total score); *NM* Negative Mood; *PM* Positive Mood; *HADS-D* Hospital Anxiety and Depression Scale–Depression subscale; *CSSS* Chronic Stress Screening Scale; *EDSS* Expanded Disability Status Scale

^a^Person-mean averages

^b^RRMS group only

### Extent of fatigue fluctuations

**Fig. 1:**
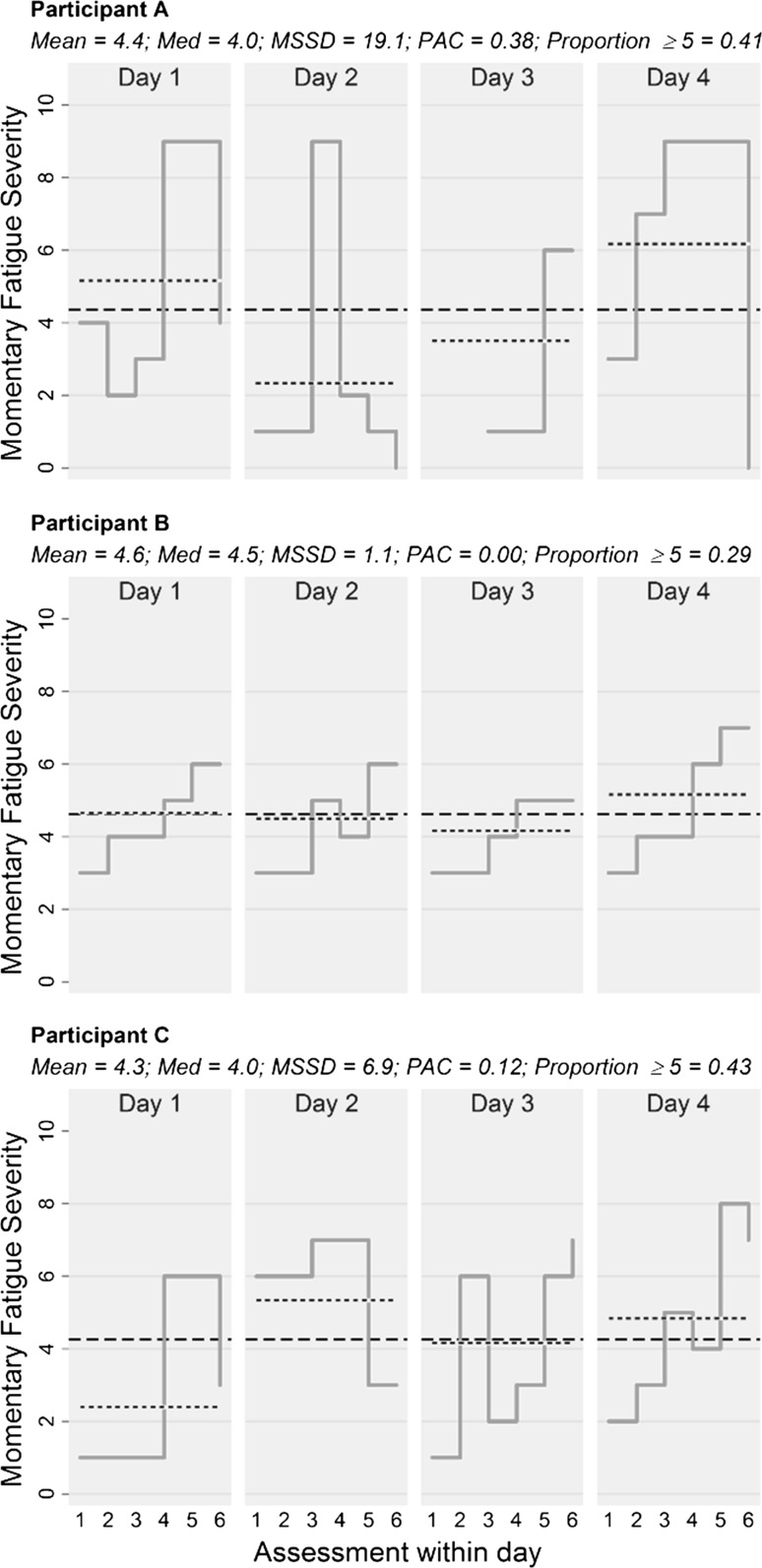
Step line charts (solid lines) depicting change in *Momentary Fatigue Severity* ratings in three individuals from the relapsing-remitting MS group over the six assessments (A1–A6) from four assessment days. Dashed lines indicate person-means and dotted lines indicate daily-means. Corresponding within-person indices are presented, including mean, median (med), mean successive squared difference (MSSD; Jahng et al., 2008), probability of acute change (PAC; acute change defined as change ≥5 units between two adjacent assessments; Jahng et al., 2008), and proportion of ratings ≥5

In the relapsing-remitting MS group, 35.2% of all observed variability in fatigue severity was attributed to moment-to-moment fluctuations, 8.2% to day-to-day changes, and 56.6% to individual differences. This was relatively similar to the 43.5% (moment-to-moment), 14.1% (day-to-day), and 42.3% (individual differences) in controls. To demonstrate the potential utility and unique information provided by within-person outcomes computed from real-time data, Fig. 1 presents single-case data from three individuals with relapsing-remitting MS with similar mean ratings but vastly different patterns of fatigue indicated by respective within-person patient reported outcomes (Jahng et al., 2008; Stone et al., 2012).

### Typical diurnal fatigue pattern

**Fig. 2:**
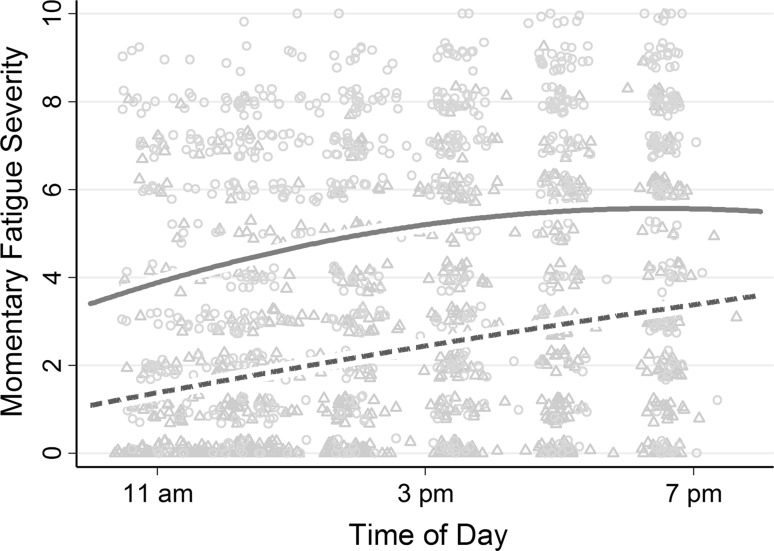
Average fatigue trajectories over time in the relapsing-remitting MS group (*red solid line*) and the control group (*green dashed line*). The circular indicators represent unique *Momentary Fatigue Severity* assessments in the relapsing-remitting MS group; the *triangular indicators* represent those in the control group

Table 4 shows *Momentary Fatigue Severity* ratings were, on average, 1.80 units higher at 10 am in the relapsing-remitting MS group than the control group (*p* < .001) after controlling for Depression subscale and Chronic Stress Screening Scale scores. *Momentary Fatigue Severity* typically increased with time in both groups, but with different temporal patterns (see Fig. 2): in relapsing-remitting MS, fatigue increased, on average, by 0.49 units per hour (linear effect; *p* < .001) but simultaneously decreased by 0.03 units per hour squared (quadratic effect; *p* = .012); in controls, fatigue increased by 0.27 units per hour (linear effect; *p* = .015).^1^ Random linear time effects were statistically significant indicating that, despite finding a robust typical diurnal fatigue pattern in the relapsing-remitting MS group, patterns differed substantially both from individual-to-individual and from day-to-day. The inclusion of time effects reduced residual variance such that 45.5% of moment-to-moment fatigue fluctuations across both groups were explained by time of day (42.7% in relapsing-remitting MS group only). Diurnal fatigue patterns remained substantially unchanged in a sensitivity analysis including no covariates, and also in a sensitivity analysis including employment status and disease modifying therapies as additional covariates.

**Table 4:** Model parameter estimates testing typical diurnal fatigue patterns in the relapsing-remitting MS group and control group, with 95% confidence intervals in square brackets

	Relapsing-remitting MS			Control			Group comparison		
	γ (SE)	[95% CI]	*p*	γ (SE)	[95% CI]	*p*	γ (SE)	[95% CI]	*p*
*Fixed effects*									
Intercept	3.24 (0.37)	[2.50, 3.98]	<.001	1.44 (0.37)	[0.70, 2.18]	<.001	1.80 (0.53)	[0.75, 2.84]	<.001
Time	0.49 (0.11)	[0.27, 0.71]	<.001	0.27 (0.11)	[0.05, 0.49]	.015	0.22 (0.16)	[−0.10, 0.53]	.177
Time^2^	−0.03 (0.01)	[−0.05, −0.01]	.012	−0.002 (0.01)	[−0.02, 0.02]	.83	−0.02 (0.02)	[−0.06, 0.01]	.10
HADS-D	0.03 (0.13)	[−0.22, 0.28]	.84	0.07 (0.14)	[−0.21, 0.35]	.62	−0.04 (0.19)	[−0.42, 0.33]	.82
CSSS	0.10 (0.03)	[0.04, 0.16]	.002	0.07 (0.04)	[−0.01, 0.15]	.070	0.03 (0.05)	[−0.07, 0.13]	.59
*Random effects*									
Level-3 (Individual)									
Intercept	2.44 (0.51)	[1.62, 3.67]	<.001						
Time	0.03 (0.01)	[0.02, 0.06]	<.001						
Level-2 (day)									
Intercept	3.16 (0.74)	[2.00, 5.00]	<.001						
Time	0.67 (0.16)	[0.42, 1.06]	<.001						
Time^2^	0.01 (0.002)	[0.004, 0.01]	<.001						
Level-1 (Assessment)									
Residual	1.53 (0.08)	[1.39, 1.69]	<.001						

*HADS-D* Hospital Anxiety and Depression Scale–Depression subscale; *CSSS* Chronic Stress Screening Scale. HADS-D and CSSS are grand-mean centred. Time is centred about 10 am. Level-3 random covariance parameters (unstructured) not presented here, but included in the model

### Contextual correlates in daily life

Table 5 shows physical exertion in the prior 30 min was associated with an average 1.00-unit increase in *Momentary Fatigue Severity* in the relapsing-remitting MS group (*p* < .001) but was not associated with *Momentary Fatigue Severity* in controls. Sleep quality was not associated with *Momentary Fatigue Severity* in the relapsing-remitting MS group, but in controls, when sleep quality was 1 SD lower than the person-mean (i.e., than usual for that person), there was an average 0.30-unit increase in *Momentary Fatigue Severity* (*p* < .001). Statistically significant interaction effects with group were evident for both physical exertion and sleep quality (*p*s < .05) indicating substantial between-group differences.

**Table 5:** Model fixed effect parameter estimates of within-person behavioural and psychosocial contextual effects with 95% confidence intervals in square brackets

	Relapsing-remitting MS			Control			Group comparison		
	γ (SE)	[95% CI]	*p*	γ (SE)	[95% CI]	*p*	γ (SE)	[95% CI]	*p*
*Model B*—*Behavioural*									
Physical exertion	1.00 (0.21)	[0.58, 1.42]	<.001	0.23 (0.24)	[−0.24, 0.70]	.33	0.77 (0.32)	[0.14, 1.39]	.017
Sleep quality	−0.02 (0.04)	[−0.11, 0.06]	.59	−0.18 (0.05)	[−0.29, −0.07]	.001	0.16 (0.07)	[0.02, 0.29]	.028
*Model C*—*Stressors*									
Work overload	0.02 (0.03)	[−0.03, 0.08]	.38	−0.02 (0.02)	[−0.06, 0.03]	.52	0.04 (0.04)	[−0.03, 0.11]	.28
Social overload	0.03 (0.03)	[−0.03, 0.09]	.31	0.03 (0.02)	[−0.02, 0.07]	.26	0.001 (0.04)	[−0.07, 0.07]	.99
Excessive demands at work	0.02 (0.03)	[−0.04, 0.09]	.47	0.06 (0.04)	[−0.02, 0.13]	.14	−0.03 (0.05)	[−0.13, 0.07]	.54
Lack of social recognition	0.08 (0.04)	[0.01, 0.15]	.025	0.09 (0.05)	[−0.002, 0.19]	.056	−0.01 (0.06)	[−0.13, 0.11]	.84
Work discontent	0.06 (0.03)	[0.001, 0.12]	.046	0.11 (0.03)	[0.05, 0.16]	<.001	−0.05 (0.04)	[−0.13, 0.03]	.25
Social tensions	0.01 (0.03)	[−0.06, 0.08]	.71	0.02 (0.04)	[−0.06, 0.10]	.60	−0.01 (0.05)	[−0.11, 0.09]	.89
Pressure to perform	−0.02 (0.03)	[−0.07, 0.03]	.46	0.01 (0.02)	[−0.04, 0.06]	.71	−0.03 (0.04)	[−0.10, 0.04]	.43
Social isolation	0.01 (0.03)	[−0.04, 0.06]	.64	−0.01 (0.03)	[−0.05, 0.04]	.83	0.02 (0.04)	[−0.05, 0.09]	.63
*Model D*—*Mood*									
Negative mood	0.17 (0.05)	[0.09, 0.26]	<.001	0.23 (0.05)	[0.13, 0.34]	<.001	−0.06 (0.07)	[−0.20, 0.08]	.42
Positive mood	−0.37 (0.04)	[−0.46, −0.28]	<.001	−0.35 (0.05)	[−0.45, −0.25]	<.001	−0.02 (0.07)	[−0.15, 0.11]	.78

Model intercepts and fixed effects of time, time^2^, Hospital Anxiety and Depression Scale–Depression subscale score, Chronic Stress Screening Scale score, and the random effects of intercept, time, and time^2^ are not shown

In the relapsing-remitting MS group, both *Lack of Social Recognition* and *Work Discontent* scores were associated within-individuals with *Momentary Fatigue Severity* such that when either stressor type was higher than usual, subsequent severity was increased (*p*s < .05; see Table 5). These two stressors showed similar effects in controls, with no significant group differences (see Table 5). Of the remaining six stressors, none were associated with *Momentary Fatigue Severity* in either group. Random effects of *Lack of Social Recognition* (*p* = .44) and *Work Discontent* (*p* = .13) did not reach statistical significance, indicated their effects were relatively consistent across individuals.

In the relapsing-remitting MS group, *Negative Mood* and *Positive Mood* was associated within-individuals with increased *Momentary Fatigue Severity* such that severity was higher when levels of negative mood were higher and when levels of positive mood were lower (*p* < .001; see Table 5). Similar associations of *Negative Mood* and *Positive Mood* with *Momentary Fatigue Severity* were present in the control group (*p*s < .001) with no statistically significant group differences in these associations (see Table 5). The random effects of *Negative Mood* (*p* = .033) and *Positive Mood* (*p* = .001) indicated substantial variability in the size of these associations across individuals.

## Discussion

As expected, substantial moment-to-moment and day-to-day fluctuations in fatigue severity were found in relapsing-remitting MS. Analysis of typical diurnal fatigue patterns found that, in relapsing-remitting MS diurnal fatigue patterns charted a quicker increase in severity in the earlier part of the day than controls, peaking in late-afternoon. Notable differences between individual diurnal patterns were evident, meaning the described pattern did not replicate across every person with relapsing-remitting MS. Healthy individuals generally exhibited a slower, steadier, accumulation of fatigue across the day. Fatigue in relapsing-remitting MS appears not only higher, but also seems to peak earlier in the day, than healthy individuals. In line with our other hypotheses, increased stressor exposure (specifically, discontent with current work activity, and lack of social recognition), increased negative mood, and decreased positive mood were all associated with increases in fatigue in real-time.

In the relapsing-remitting MS group, we found reasonably strong associations of both person-mean real-time fatigue severity and daily fatigue severity with Chalder Fatigue Questionnaire scores. However, we have demonstrated that patient reported outcomes based, implicitly or explicitly, on mean average severity, overlook potentially-important information about time- and context-dependent fluctuations. Real-time data can provide informative indicators of symptom experience to complement the mean average (Stone et al., 2012). Future trials could consider demonstrating treatment efficacy by identifying and alleviating those aspects of the overall ‘fatigue experience’ deemed most important by the individual (Stone et al., 2012). For some individuals, one or more inherently within-person facets of fatigue severity may be of greatest relevance to quality of life; speculatively, acutely-fluctuating symptoms may cause considerably more uncertainty (and hinder adaptive adjustment) than stable symptoms.

Fatigue appeared to generally peak in late-afternoon in the relapsing-remitting MS group, corroborating earlier qualitative studies (Freal et al., 1984; Mills & Young, 2008). A ceiling effect was considered unlikely here given the maximum *Momentary Fatigue Severity* rating was infrequently used (<3%). Future research may identify trait or state factors predicting deviations from typical diurnal fatigue patterns. The present study suggested relapsing-remitting MS fatigue is not affected by daily changes in sleep quality, which was surprising given a recent review found sleep problems in MS contribute to fatigue (Strober, 2015). Given that there is a wealth of literature demonstrating robust increases in fatigue after a night of sleep deprivation in other clinical conditions (Irwin et al., 2012; Nicassio et al., 2002), future studies will need to test this temporal relationship using an objective measure of sleep continuity such as polysomnography. A period of physical exertion increased fatigue in the relapsing-remitting MS group, resembling post-exertional malaise: an important symptom in chronic fatigue syndrome that is, broadly, an acute increase in fatigue (and other symptoms) following exertion that has an extended recovery time (Carruthers et al., 2011; Fukuda et al., 1994). However, the exertion measure in the present study was binary (yes/no) with no detail about intensity. There is some evidence, albeit inconsistent across studies, that physical activity has beneficial effects on MS fatigue (Latimer-Cheung et al., 2013) and a Cochrane review concluded that there is an overall moderate effect of exercise therapy on reducing fatigue (Heine et al., 2015). Further research incorporating objective measures of activity are required to precisely elucidate the within-person effect of exertion on fatigue in MS, and to further explore similarities with post-exertional malaise.

The present study adds a within-person perspective to existing studies demonstrating associations between stress and fatigue in MS (Trojan et al., 2007). Individuals in both groups were more fatigued after periods in which they felt discontented with their current work, or underappreciated for their efforts. Of the eight stressor types measured, the two items associated with fatigue (*Lack of Social Recognition*; *Work Discontent*) are conceptually linked with one’s motivation to persist with the current task. These findings support theoretical developments suggesting general fatigue is an emotional experience prompting a (likely unconscious) re-evaluation of the costs and benefits of continuing with the present activity, and a redirection of attention toward other behaviors with greater utility (Hockey, 2013; Inzlicht et al., 2014; Kurzban et al., 2013). Crucially, we found no evidence that these stressors, or mood, had magnified within-person effects on fatigue in relapsing-remitting MS.

In chronic fatigue syndrome, positive correlations ranging from small to moderate in size have been found between person-mean momentary fatigue intensity and person-mean negative affect, depression, anxiety, and catastrophizing (Sohl & Friedberg, 2008). In the present study, person-mean correlations between negative mood and fatigue severity were not statistically significant in the relapsing-remitting MS group, but a large correlation was found in the control group, again indicating that MS-related fatigue is a different phenomenon to the fatigue experienced by healthy individuals. This was despite average levels of negative mood being higher in the MS group than controls. Moderate and negative correlations with person-mean positive mood were evident in both groups, and was not experienced less frequently in the relapsing-remitting MS group.

The described real-time contextual associations do not infer direct causality (although stressors measured ‘since the last event’ imply pooled stressors over that period occurred before fatigue ‘right now’) and a lagged-effects analysis with a more-intensive ecological momentary assessment schedule may further explain directions of effects.

The limited reliability of the stressor factor scores was likely due to the low frequency of stressors observed, resulting in heavily skewed distributions, and the relatively small number of items contributing to each factor. Multiple testing with individual stressor items increased the risk of spurious findings, but was considered unlikely here given statistically significant effects were consistent across groups. Nevertheless, the exploratory nature of these stressor effects is emphasized.

Participant compliance was excellent, with few missing assessments. The quasi-random design minimized prompt anticipation, and yielded a representative sample of daily living (Broderick et al., 2008). Prompts between 10 am and 8 pm were chosen to limit the possibility that fatigue measures would be confounded by sleepiness, considered a distinct phenomenon (Shen et al., 2006). However, understanding of early-morning fatigue is therefore limited. Weekday-weekend differences in fatigue may also be worthy of investigation in future studies in MS. The recruited sample was relatively homogeneous, with no comorbidities, and most were still full-time employed. Further investigations could explore the generalizability of the findings to people with relapsing-remitting MS and common comorbidities, such as depression, and to people with progressive MS-types. Future studies may also compare fatigue trajectories in MS to those found in other conditions with characteristic chronic fatigue, such as cancer or fibromyalgia, or to chronic fatigue syndrome itself.

The study has some limitations. A concern with ecological momentary assessment studies is that intensive self-monitoring may change the experience of the symptom being monitored: a process known as measurement reactivity (Barta et al., 2012). While there is no or negligible evidence of measurement reactivity in many empirical investigations of the phenomenon (for example, Aaron et al., 2005; Peters et al., 2000; Sonnenschein et al., 2006; Stone et al., 2003) it has been noted that more work is needed to explore this phenomenon in other domains, including fatigue (Barta et al., 2012). Here, *Momentary Fatigue Severity* combined physical with mental fatigue into a single item measuring general fatigue severity. It may be informative for future studies to examine physical and mental fatigue in daily life separately; however, a recent psychometric analysis of the Chalder Fatigue Questionnaire in MS found one general fatigue factor accounted for 81.4% of variance in a bi-factor model, suggesting a limited practical distinction between physical and mental fatigue constructs (Chilcot et al., 2015).

This study is the first prospective investigation of temporal and contextual effects on real-time fatigue severity in relapsing-remitting MS: typically, fatigue increased over the day but decelerated toward a peak in late-afternoon, while contextual associations with specific stressors and mood were evident. Findings suggest future MS fatigue interventions could explore ways of improving positive mood and responding to interpersonal and work stressors differently. Ways to manage peak fatigue in the afternoon and after physical exertion should be explored, possibly using scheduled rest breaks or short naps (less than 30 min) in accordance with current guidance (Multiple Sclerosis Council for Clinical Practice Guidelines, 2000). Notably, temporal effects and associations with momentary mood varied substantially across individuals. Increasing our understanding of how fatigue is dynamically experienced by each individual may present opportunities to further develop tailored interventions targeting fatigue.

## Electronic supplementary material

Below is the link to the electronic supplementary material.

Supplementary material 1 (DOCX 16 kb)

Supplementary material 2 (DOCX 18 kb)

Supplementary material 3 (DOCX 23 kb)

## Footnotes

1 Prompted by a reviewer, we examined whether people in the MS group were more likely to nap in the afternoon, explaining the negative quadratic effect. Naps (*n* = 24 in the MS group; *n* = 8 in the control group) were not more likely later in the day in either group. The linear and quadratic effects reported also persisted when including naps in the model.
