# Supplementary material for: Tracking daily fatigue fluctuations in multiple sclerosis: ecological momentary assessment provides unique insights
Source: J Behav Med. 2017 Mar 9;40(5):772–83. doi: 10.1007/s10865-017-9840-4 (PMC5613039; doi:10.1007/s10865-017-9840-4)
Supplement: Supplementary file 1 — Supplementary material 1 (DOCX 16 kb) [file 10865_2017_9840_MOESM1_ESM.docx]

Supplementary 1

Table S1. Study’s ecological momentary assessment (EMA) schedule

| Schedule | Items | Construct being measured |
| --- | --- | --- |
| Momentary Assessments  A1 – A6  (10am – 8pm) | Prefix: *Since the last event…*  I did a lot of work  I dealt a lot with other people’s matters  I performed some of my tasks inadequately  Others undervalued my work  I felt discontented with the type of work Im doing  I had a disagreement with someone  I performed tasks that allowed no mistakes  It was important to ensure good relations with another person  Response format: *0 (Not at all) to 10 (Very much so)*  How much fatigue (weariness, tiredness, problems thinking clearly) do you feel right now?  Response format: *0 (None at all) to 10 (Extreme Fatigue)*  Prefix: *At the moment, I feel…*  Distressed  Upset  Guilty  Irritable  Ashamed  Anxious  Calm [reverse-scored]  Down  Worried  Angry  Response format: *0 (Not at all) to 10 (Very much so)*  Prefix: *At the moment, I feel…*  Proud  Alert  Determined  Energetic  Satisfied  Response format: *0 (Not at all) to 10 (Very much so)*  Prefix: *In the last 30 minutes, have you been…*  Exerting physically?  Eating?  Sleeping (napping)?  Drinking coffee?  Response format: *Yes or No* | Momentary Stressor Exposure  Work Overload  Social Overload  Excessive Demands at Work  Lack of Social Recognition  Work Discontent  Social Tensions  Pressure to Perform  Social Isolation  Momentary Fatigue Severity  Negative Affect subscale  Positive Affect subscale  Behavioural Factors |
| Recall Assessment  A7  (9pm) | How much fatigue (weariness, tiredness, problems thinking clearly) have you felt today?  Response format: *0 (None at all) to 10 (Extreme Fatigue)* | Daily Fatigue Severity |

Note. A1 – A6 denotes quasi-randomly presented prompts placed within each of six consecutive 100 min time-windows. A7 denotes the single fixed-time prompt at 9pm. For details about the exploratory factor analysis carried out for the affect items, see Supplementary 2.
