# Supplementary material for: Tracking daily fatigue fluctuations in multiple sclerosis: ecological momentary assessment provides unique insights
Source: J Behav Med. 2017 Mar 9;40(5):772–83. doi: 10.1007/s10865-017-9840-4 (PMC5613039; doi:10.1007/s10865-017-9840-4)
Supplement: Supplementary file 2 — Supplementary material 2 (DOCX 18 kb) [file 10865_2017_9840_MOESM2_ESM.docx]

Supplementary 2

The two exploratory factor analyses (EFA) presented below were conducted with MPlus 7.3.

**EFA1: Stress**

Table S2A. Stressor items presented within EMA schedule.

| Eight items, prefixed by ‘Since the last event….’  Response: 0 (Not at all) – 10 (Very much so) |
| --- |
| I did a lot of work  I dealt a lot with other people’s matters  I performed some of my tasks inadequately  Others undervalued my work  I felt discontented with the type of work Im doing  I had a disagreement with someone  I performed tasks that allowed no mistakes  It was important to ensure good relations with another person |

Eight stressor items were presented (see Table A) based on the eight domains of the Trier Inventory of Chronic Stress.^1^ Each item was prefixed with ‘Since the last event…’ with response sliders from 0 ‘Not at all’ to 10 ‘Very much so’. An exploratory factor analysis (EFA) for categorical outcome variables with cluster-robust computations of standard errors and chi-squared statistics to account for repeated measurements within subjects was conducted using Mplus 7.3. For the EFA, stressor responses were categorized into five categories to account for non-normality of their distributions. The results suggested a 3-factor solution with good model fit, χ² = 12.3; df = 7; *p* = .09; Root Mean Square Error of Approximation (RMSEA) = .021; *p* (RMSEA ≤ .05) = .99; Comparative Fit Index (CFI) = .99; Tucker-Lewis-Index (TLI) = .97. Four items loaded onto a major factor (items: ‘others undervalued my work’; ‘I performed some of my tasks inadequately’; ‘I had a disagreement with someone’; ‘I felt discontented with the type of work I was doing’). The remaining two factors were less clear, so were discarded. A Daily Life Stress scale score was computed as the mean average of the four constituent items, but estimation of its composite within-subject reliability^2^ showed limited reliability (ω_within_ = .48). Due to this limited reliability, this factor was also discarded and we decided to use the individual stressor items as predictors, in an exploratory analysis.

**EFA2: Affect**

Table S2B. Affect items presented within EMA schedule

| Fifteen items, prefixed by ‘At the moment, I feel….’  Response: 0 (Not at all) – 10 (Very much so) |
| --- |
| Distressed  Upset  Guilty  Irritable  Ashamed  Anxious  Calm  Down  Worried  Angry |

Momentary affect was measured with 15 mood adjectives, prefixed by ‘At the moment, I feel…’ with response sliders from 0 ‘Not at all’ to 10 ‘Very much so’. A cluster-robust EFA for categorical outcome variables (using the same cut-off values as for the stressor items) yielded a 2-factor solution, with acceptable model fit, χ² = 333.7; df = 76; p < .001; RMSEA = .045; p (RMSEA ≤ .05) = .94; CFI = .94; TLI = .92. Ten items loaded onto one factor, ‘Negative Affect’ (items: ‘distressed’, ‘upset’, ‘guilty’, ‘irritable’, ‘ashamed’, ‘anxious’, ‘calm’ [reverse scored], ‘down’, ‘worried’, ‘angry’) and five items loaded onto another factor, ‘Positive Affect’ (items: ‘proud’, ‘alert’, ‘determined’, ‘energetic’, ‘satisfied’). Scale scores were computed as the mean of constituent items and demonstrated satisfactory within-subject reliability of ω_within_ = .86 for Negative Affect and ω_within_ = .68 for Positive Affect.

**References**

1. Schulz P, Schlotz W, Becker P. *Trierer Inventar zum Chronischen Stress (TICS) [Trier Inventory for Chronic Stress (TICS)]*. Gottingen: Hogrefe Verlag, 2004.

2. Geldhof GJ, Preacher KJ, Zyphur MJ. Reliability estimation in a multilevel confirmatory factor analysis framework. Psychological Methods 2014;**19**(1):72-91.
