# Supplementary material for: Tracking daily fatigue fluctuations in multiple sclerosis: ecological momentary assessment provides unique insights
Source: J Behav Med. 2017 Mar 9;40(5):772–83. doi: 10.1007/s10865-017-9840-4 (PMC5613039; doi:10.1007/s10865-017-9840-4)
Supplement: Supplementary file 3 — Supplementary material 3 (DOCX 23 kb) [file 10865_2017_9840_MOESM3_ESM.docx]

Supplementary 3

**Technical details of multilevel models used in the study**

All specified models use standard notation^1^.To test typical diurnal (daily) fatigue patterns, linear and quadratic models of *Momentary Fatigue Severity* were explored for best fit by chi square tests on the deviance statistic (difference in -2 Log Likelihood). Consecutive growth models were built using all data, adding complexity with each step, until a random quadratic covariate growth model was derived as the best fit (see Equation 1 below; Model A). This final model had an unstructured variance-covariance matrix to model the residuals at level-2, and a diagonal matrix modelling residuals at level-3. Group differences in diurnal fatigue patterns were examined by adding group as a binary fixed effect at level-3 with group by time cross-level interaction effects.

As stated in the manuscript, all subsequent models are based on Model A with the addition of predictors of interest.

**Equation S1 (Model A)** - Random quadratic covariate growth model.

$${FATIGUE}_{adi}= \gamma_{000}+ \gamma_{100}\left( {TIME}_{adi} \right)+ \gamma_{200}\left( {TIME}_{adi} \right)^{2}+ \gamma_{001}\left( {DEPRESSIVE SYMPTOMS}_{i} \right)+ \gamma_{002}\left( {CHRONIC STRESS}_{i} \right)+ V_{00i}+ V_{10i}\left( {TIME}_{adi} \right)+ U_{0di}+ U_{1di}\left( {TIME}_{adi} \right)+ U_{2di}\left( {TIME}_{adi} \right)^{2}+ R_{adi}$$

Where $R_{adi}$ ~ $N(0,\sigma_{R}^{2})$ and $\left[ \begin{matrix} U_{0di} \\ U_{1di} \\ U_{2di} \end{matrix} \right]$ ~ $N\left( \left[ \begin{matrix} 0 \\ 0 \\ 0 \end{matrix} \right] , \left[ \begin{matrix} \sigma_{0}^{2} & \sigma_{01} & \sigma_{02} \\ \sigma_{10} & \sigma_{1}^{2} & \sigma_{12} \\ \sigma_{20} & \sigma_{21} & \sigma_{2}^{2} \end{matrix} \right] \right)$ and $\left( \begin{matrix} V_{00i} \\ V_{10i} \end{matrix} \right)$ ~ $N\left( \left[ \begin{matrix} 0 \\ 0 \end{matrix} \right] , \left[ \begin{matrix} \sigma_{00}^{2} & 0 \\ 0 & \sigma_{10}^{2} \end{matrix} \right] \right)$

Where ${FATIGUE}_{adi}$ is the value of Momentary Fatigue Severity for individual $i$ on day $d$ at assessment $a$, ${TIME}_{adi}$ is the time of day for individual $i$ on day $d$ at assessment $a$, ${DEPRESSIVE SYMPTOMS}_{i}$ is the level of depressive symptoms (HADS-D score) for individual $i$, and ${CHRONIC STRESS}_{i}$ is the rating of chronic stress (CSSS score) for individual $i$. Fixed effects are denoted by $\gamma$, with $\gamma_{000}$ indicating the average intercept, $\gamma_{100}$ the average effect of ${TIME}_{adi}$, $\gamma_{200}$ the average effect of ${{TIME}_{adi}}^{2}$, $\gamma_{001}$ the average effect of ${DEPRESSIVE SYMPTOMS}_{i}$, and$\gamma_{002}$ the average effect of ${CHRONIC STRESS}_{i}$ . Random effects at level-3 (individual) are indicated by $V$, with $V_{00i}$ denoting the deviation of the intercept for individual $i$ from the average intercept, $V_{10i}$ denoting the deviation of the $TIME$ effect for individual $i$ from the average effect of $TIME$. Random effects at level-2 (day) are indicated by $U$, with $U_{0di}$ denoting the deviation of the intercept on day $d$ from the average intercept for individual $i$, $U_{1di}$ denoting the deviation of the $TIME$ effect for day $d$ from the average effect of $TIME$ of individual $i$, and $U_{2di}$ denoting the deviation of the $TIME$^2^ effect for day $d$ from the average effect of $TIME$^2^ for individual $i$. $R_{adi}$ indicates the model residuals at level-1 (assessments).

The remaining models are presented below in equation form:

**Equation S2 (Model B)** – Testing the effects of physical exertion and sleep quality.

$${FATIGUE}_{adi}= \gamma_{000}+ \gamma_{100}\left( {TIME}_{adi} \right)+ \gamma_{200}\left( {TIME}_{adi} \right)^{2}+ \gamma_{001}\left( {DEPRESSIVE SYMPTOMS}_{i} \right)+ \gamma_{002}\left( {CHRONIC STRESS}_{i} \right)+ \gamma_{300}\left( {EXERTION}_{adi} \right)+ \gamma_{010}\left( {SLEEP QUALITY}_{di} \right)+ V_{00i}+ V_{10i}\left( {TIME}_{adi} \right)+ U_{0di}+ U_{1di}\left( {TIME}_{adi} \right)+ U_{2di}\left( {TIME}_{adi} \right)^{2}+ R_{adi}$$

Where $R_{adi}$ ~ $N(0,\sigma_{R}^{2})$ and $\left[ \begin{matrix} U_{0di} \\ U_{1di} \\ U_{2di} \end{matrix} \right]$ ~ $N\left( \left[ \begin{matrix} 0 \\ 0 \\ 0 \end{matrix} \right] , \left[ \begin{matrix} \sigma_{0}^{2} & \sigma_{01} & \sigma_{02} \\ \sigma_{10} & \sigma_{1}^{2} & \sigma_{12} \\ \sigma_{20} & \sigma_{21} & \sigma_{2}^{2} \end{matrix} \right] \right)$ and $\left( \begin{matrix} V_{00i} \\ V_{10i} \end{matrix} \right)$ ~ $N\left( \left[ \begin{matrix} 0 \\ 0 \end{matrix} \right] , \left[ \begin{matrix} \sigma_{00}^{2} & 0 \\ 0 & \sigma_{10}^{2} \end{matrix} \right] \right)$

*Note:* Fixed effect of group and group interactions added to the model to detect group differences and average within-group effects.

**Equation S3 (Model C)** – Testing the independent effects of each stressor type.

$${FATIGUE}_{adi}= \gamma_{000}+ \gamma_{100}\left( {TIME}_{adi} \right)+ \gamma_{200}\left( {TIME}_{adi} \right)^{2}+ \gamma_{001}\left( {DEPRESSIVE SYMPTOMS}_{i} \right)+ \gamma_{002}\left( {CHRONIC STRESS}_{i} \right)+ \gamma_{300}\left( {WORK OVERLOAD}_{adi} \right)+ \gamma_{400}\left( {SOCIAL OVERLOAD}_{adi} \right)+ \gamma_{500}\left( {EXCESSIVE DEMANDS}_{adi} \right)+ \gamma_{600}\left( {LACK OF SOCIAL RECOGNITION}_{adi} \right)+ \gamma_{700}\left( {WORK DISCONTENT}_{adi} \right)+ \gamma_{800}\left( {SOCIAL TENSIONS}_{adi} \right)+ \gamma_{900}\left( {PRESSURE TO PERFORM}_{adi} \right)+ \gamma_{10;00}\left( {SOCIAL ISOLATION}_{adi} \right)+ V_{00i}+ V_{10i}\left( {TIME}_{adi} \right)+ U_{0di}+ U_{1di}\left( {TIME}_{adi} \right)+ U_{2di}\left( {TIME}_{adi} \right)^{2}+ R_{adi}$$

Where $R_{adi}$ ~ $N(0,\sigma_{R}^{2})$ and $\left[ \begin{matrix} U_{0di} \\ U_{1di} \\ U_{2di} \end{matrix} \right]$ ~ $N\left( \left[ \begin{matrix} 0 \\ 0 \\ 0 \end{matrix} \right] , \left[ \begin{matrix} \sigma_{0}^{2} & \sigma_{01} & \sigma_{02} \\ \sigma_{10} & \sigma_{1}^{2} & \sigma_{12} \\ \sigma_{20} & \sigma_{21} & \sigma_{2}^{2} \end{matrix} \right] \right)$ and $\left( \begin{matrix} V_{00i} \\ V_{10i} \end{matrix} \right)$ ~ $N\left( \left[ \begin{matrix} 0 \\ 0 \end{matrix} \right] , \left[ \begin{matrix} \sigma_{00}^{2} & 0 \\ 0 & \sigma_{10}^{2} \end{matrix} \right] \right)$

*Note:* Fixed effect of group and group interactions added to the model to detect group differences and average within-group effects.

**Equation S4 (Model D)** – Testing the independent effects of negative and positive affect.

$${FATIGUE}_{adi}= \gamma_{000}+ \gamma_{100}\left( {TIME}_{adi} \right)+ \gamma_{200}\left( {TIME}_{adi} \right)^{2}+ \gamma_{001}\left( {DEPRESSIVE SYMPTOMS}_{i} \right)+ \gamma_{002}\left( {CHRONIC STRESS}_{i} \right)+ \gamma_{300}\left( {NEGATIVE AFFECT}_{adi} \right)+ \gamma_{400}\left( {POSITIVE AFFECT}_{adi} \right) + V_{00i}+ V_{10i}\left( {TIME}_{adi} \right)+ U_{0di}+ U_{1di}\left( {TIME}_{adi} \right)+ U_{2di}\left( {TIME}_{adi} \right)^{2}+ R_{adi}$$

Where $R_{adi}$ ~ $N(0,\sigma_{R}^{2})$ and $\left[ \begin{matrix} U_{0di} \\ U_{1di} \\ U_{2di} \end{matrix} \right]$ ~ $N\left( \left[ \begin{matrix} 0 \\ 0 \\ 0 \end{matrix} \right] , \left[ \begin{matrix} \sigma_{0}^{2} & \sigma_{01} & \sigma_{02} \\ \sigma_{10} & \sigma_{1}^{2} & \sigma_{12} \\ \sigma_{20} & \sigma_{21} & \sigma_{2}^{2} \end{matrix} \right] \right)$ and $\left( \begin{matrix} V_{00i} \\ V_{10i} \end{matrix} \right)$ ~ $N\left( \left[ \begin{matrix} 0 \\ 0 \end{matrix} \right] , \left[ \begin{matrix} \sigma_{00}^{2} & 0 \\ 0 & \sigma_{10}^{2} \end{matrix} \right] \right)$

*Note:* Fixed effect of group and group interactions added to the model to detect group differences and average within-group effects.

**References**

1. Snijders TAB, Bosker RJ. *Multilevel Analysis: An Introduction to Basic and Advanced Multilevel Modeling*. 2nd Edition ed. London, UK: SAGE Publications, 2012.
